# Hazard Assessment of Ag Nanoparticles in Soil Invertebrates—Strong Impact on the Longer-Term Exposure of *Folsomia candida*

**DOI:** 10.3390/jox15060210

**Published:** 2025-12-12

**Authors:** Susana I. L. Gomes, Janeck J. Scott-Fordsmand, Mónica J. B. Amorim

**Affiliations:** 1 Department of Biology & CESAM, University of Aveiro, 3810-193 Aveiro, Portugal; mjamorim@ua.pt; 2 Department of Ecoscience, Aarhus University, C.F. Møllers Alle 4, DK-8000 Aarhus, Denmark

**Keywords:** nanomaterials, prolonged exposure, multi-generation, collembolans

## Abstract

Silver nanomaterials (Ag NMs) are widely used, including in consumer products, and they inevitably enter the environment, with the soil compartment acting as a major sink. However, most available toxicity data focus on the reference Ag NM300K and rely on standard tests, even though long(er)-term exposure tests are recognized as particularly important for assessing the risks to soil invertebrates. Hence, the aim of the present study was to investigate the toxicity of commercial Ag NPs (Ag-Sigma, NPs < 150 nm) to the soil ecotoxicology model *Folsomia candida* (Collembola). Effects were assessed based on the standard OECD reproduction test (28 days) and beyond, with exposure prolonged for a second generation (56 days). Results showed that, based on the standard test (50% reproduction effect concentration—EC50 = 988 mg Ag/kg soil), the commercial Ag NPs were less toxic than the reference Ag NM300K and the ionic form AgNO_3_ (from literature). However, the toxicity dramatically increased (ca. 4 times) during the second-generation exposure (EC50(56d) = 234 mg Ag/kg soil), surpassing the toxicity of Ag NM300K. The decrease in adults’ size indicates that moulting might be affected. Overall, increased toxicity in prolonged exposure was not expected based on the available and standard test results, which highlights the importance of long(er)-term exposures to fully assess the risks of NMs to soil communities.

## 1. Introduction

Silver nanomaterials/nanoparticles (Ag NMs/NPs) (the terms NMs and NPs will be used interchangeably throughout this paper) are among the most used NMs worldwide, with applications ranging from consumer products (e.g., cosmetics, textiles, food packaging) and electronics to (bio)medical applications and biocides. Recent reports found that the global silver nanoparticles (Ag NPs) market size surpassed USD 3000 million (more than EUR 2500 million) in 2024 [1] and is estimated to grow at more than 10% up to 2033 [1,2]. Hence, the likelihood is high for an increase in Ag NPs reaching the environment. Regarding the soil compartment, direct application is among the entry routes for Ag NMs, either in the form of biocides or biosolids (e.g., sewage sludge from wastewater treatment plants (WWTPs) [3,4]. A probabilistic material flow analysis study, specifically applied to China production and environmental information for Ag NMs, predicted that >90% of Ag NMs in commercial products end up in WWTPs [5]. Another study considering the European waste treatment systems indicated that (in 2020) soils were the environmental compartment receiving the highest load of nano-Ag [6]. Hence, the hazards of Ag NMs should be thoroughly investigated in soil ecosystems.

Over the last decade, toxicity studies have shown that Ag NMs exposure affects the survival and reproduction of some soil model invertebrate species, including the collembolan *Folsomia candida* [7,8,9,10,11], different earthworm species, including *Eisenia fetida* and *Lumbricus rubellus* [10,12,13,14,15], and the enchytraeids *Enchytraeus crypticus* and *Enchytraeus albidus* [9,16,17,18,19,20]. Avoidance behaviour was also investigated, overall showing that earthworms and enchytraeids can detect and avoid Ag NMs [10,19,21,22,23].

Mechanistic-based endpoints such as gene expression and oxidative stress biomarker analysis provided indications that Ag ions, as released from Ag NMs, play a role in NMs toxicity (e.g., [16,24,25,26,27,28]). Further, the influence of NMs’ physicochemical properties, such as size, surface coating, or shape, on toxicity has also been investigated (e.g., [9,13,14,19]) without consistent conclusions [4]. It is also known that Ag NMs undergo several transformations in media like soil [3], which can influence toxicity. For instance, the toxicity of Ag NMs was shown to increase in aged soils [11,15], which can be related to the toxicokinetics of Ag. That is, a low uptake rate constant with virtually no elimination of Ag NM suggests a constant increase in Ag body concentrations [20]. In fact, studies with other NMs/NPs also indicate that longer-term effects are often not predicted based on shorter-term exposures [29,30,31,32,33,34,35]; hence, prolonged exposures (longer than the standard tests, e.g., twice the exposure time) are recommended to assess the risks of NMs in soils.

Despite the relative abundance of toxicity data for Ag NMs in soils (even though much lower than for the aquatic environment [4]), most of the studies were performed on the JRC and standard OECD material Ag NM300K [36], a colloidal dispersion of spherical Ag NPs (10% Ag) with a nominal size of ca. 15 nm. Although the testing of standard materials is very important to ensure comparability, consistency, and reliability of results across laboratories, species, test media, etc., there are Ag materials with potential use in industry that virtually lack toxicity testing. Among the commercially available Ag NMs, an Ag nanopowder (<150 nm) by Sigma-Aldrich is being tested for use in several applications (e.g., electrolysis [37,38,39], coatings [40], antimicrobial [41,42]). However, it is sparsely tested for toxicity to soil-living organisms. One study performed with the enchytraeid *E. crypticus* showed that the Ag NPs (Ag-Sigma < 150 nm) inhibit the animals’ reproduction with a 50% effect concentration (EC50) at ca. 450 mg Ag/kg soil [18].

Given the existing knowledge gap regarding commercially available Ag NMs, this study investigated the environmental hazards of Ag NPs (Ag-Sigma < 150 nm) using the soil ecotoxicity model species *Folsomia candida* (Collembola, Arthropoda). The tests were performed in the natural standard LUFA 2.2 soil, following the standard OECD test procedures [43] (28 days) and the well-developed longer-term test that runs for an additional generation (total 56 days exposure [44]), to assess effects on animals’ survival, reproduction, size, and estimated biomass. This approach delivers toxicity data in accordance with standard OECD procedures, along with the longer-term toxicity assessments recommended for NMs, for a commercial material for which such data is not available. Hence, contributing to closing current knowledge gaps.

## 2. Materials and Methods

### 2.1. Test Species

The springtail *Folsomia candida* (Collembola) was used as the test species. Animals were cultured in plastic containers containing a moist substrate consisting of a mixture of plaster of Paris and activated charcoal (in a ratio of 8:1) at 20 ± 1 °C with a photoperiod of 16 h (8 h dark). The animals were fed weekly with granulated dried baker’s yeast (*Saccharomyces cerevisiae*). Cultures were synchronized to obtain 10–12-day-old juveniles for test start [43].

### 2.2. Test Soil

The standard LUFA 2.2 natural soil (Speyer, Germany) was used. The soil’s main characteristics are pH (0.01 M CaCl_2_) of 5.5, 1.77% organic matter, 10.1 meq/100 g CEC (cation exchange capacity), 44.8% WHC (water holding capacity), and grain size distribution of 7.3% clay, 13.8% silt, and 78.9% sand.

### 2.3. Test Materials, Characterization, and Spiking

The commercial nanomaterial Ag nanopowder (<150 nm, 99 %, CAS number 7440-22-4, Sigma-Aldrich, Merck KGaA, Darmstadt, Germany) was used and is further referred to as Ag-Sigma. The morphology of the particles was characterized based on Transmission Electron Microscopy (TEM) using a FEI TECNAI F20 instrument microscope (Hillsboro, OR, USA) operating at 200 keV, revealing agglomeration of particles within a size range in agreement with the report by the producer: crystalline particles with irregular morphology and size ranging from 10 to 150 nm. For full details, please see [18].

The tested concentrations were: 0, 32, 100, 320, 1000, and 3200 mg Ag/kg soil dry weight (DW). This wide concentration range aims to cover a full dose–response curve. Further, it is above the hazard concentrations for 50% of species (HC_50_) = 3.09 mg/kg for Ag NMs, based on species sensitivity distributions (SSDs) for soil living organisms (including microbes, invertebrates, and plants) [4].

Spiking followed the recommendations for nanomaterials [45]. In short, dry powders of Ag-Sigma were added to dry soil and vigorously mixed manually, with a spatula, for about 1 min for a homogeneous distribution of the particles in the soil. That was performed per individual replicate to obtain the corresponding concentration range. Deionised water was subsequently added to bring the soil to 50% of its maximum WHC, followed by thorough mixing. Soil was allowed to equilibrate for 1 day before the start of the test.

### 2.4. Ecotoxicity Test Procedures

The toxicity tests followed the standard OECD guideline [43] (28 days) plus its extension (56 days), representing one more generation compared to the standard, as described in Guimarães et al. [44]. Briefly, the endpoints and sampling times were (i) survival and reproduction: at 7, 14, 21, 28, and 56 days; and (ii) size and estimated biomass: at 28 and 56 days. Four replicates per treatment were performed, except on days 7, 14, and 21, with one replicate for additional monitoring. At the beginning of the test, ten synchronized age animals (10–12 days old) were placed in each test vessel (5.5 cm in diameter) containing 30 g of moist soil and food (2–10 mg of baker’s yeast). Test ran at 20 ± 1 °C, with a 16:8 h photoperiod. Weekly, food supply (2–10 mg, baker’s yeast) and water were replenished. At each sampling day, test vessels were flooded with water, carefully stirred, and all the content was transferred to a crystallizer dish. Then, the surface was photographed for further analyses (count and measure (size, area) of the floating animals) using the software ImageJ (v.1.52a, Wayne Rasband, National Institutes of Health, Bethesda, MD, USA). For the second-generation exposure (up to 56 days), at day 28, ten of the biggest sampled juveniles (ca. 11 days old) were transferred to new test vessels containing moist soil (control or spiked at day −1), representing an F1 exposure. The test ran under the same exact conditions as F0. At day 56, survival (F1) and reproduction (F2) were assessed, and the animals were measured, following the previously described procedure.

### 2.5. Data Analysis

The population biomass was calculated as based on the size of the organisms (area, in mm^2^) times the number of organisms, which is linearly correlated to animals’ volume (mm^3^). To assess differences in all the endpoints (survival, reproduction, size, and biomass) between control and treatment groups, a one-way ANOVA followed by the Dunnett’s post hoc test was used(SigmaPlot, SPSS Statistics for Windows, version 14.0 (SPSS Inc., Chicago, IL, USA)). To calculate the effect concentrations (ECx), data were modelled to the logistic or threshold sigmoid 2 parameters regression models, as indicated in Table 1, using the Toxicity Relationship Analysis Program software (TRAP v1.30a, USEPA). The *x*-axis data (concentration) was log-transformed due to improved model fitting.

## 3. Results

The validity criteria, as described in the standard OECD test guidelines [43], were met, i.e., in controls, adult mortality was below 20%, the mean number of juveniles was higher than 100, and the coefficient of variation was less than 30% for the number of juveniles. This was also the case for the extension to the OECD standard, i.e., 56 days duration.

**Figure 1:**
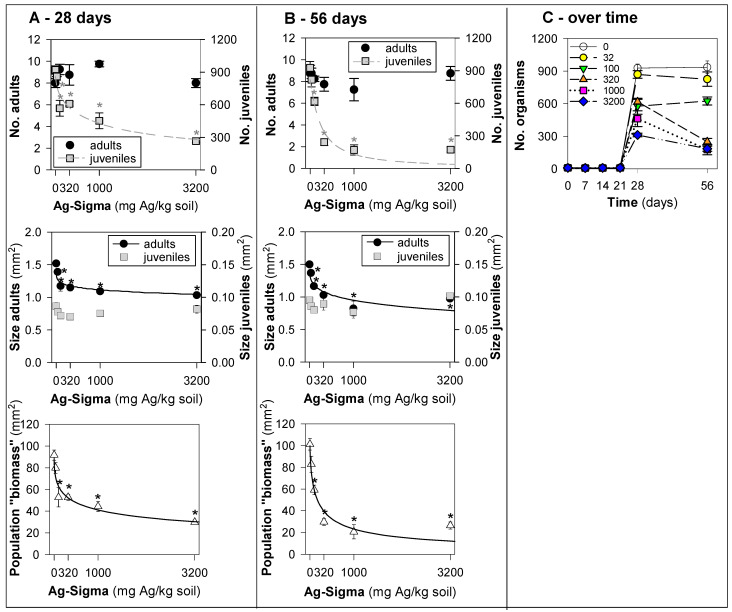
Results in terms of survival, reproduction, size, and biomass of *Folsomia candida* exposed to silver nanoparticles (Ag-Sigma) in LUFA 2.2 for (**A**) 28 days (OECD Standard), (**B**) 56 days (2nd generation), and (**C**) overview of the time series sampling at days 7, 14, 21, 28, and 56. Values represent the number of adults, juveniles, population, size (area, mm^2^), and biomass (mm^2^) as average ± standard error (AV ± SE), n = 4. *: *p* < 0.05 (Dunnett’s).

Exposure to Ag-Sigma did not significantly affect the survival of *F. candida* in either of the two exposure generations (28, 56 days) (Figure 1A,B).

Nevertheless, there was a clear impact on the size of these adults, being significantly smaller and with a dose–response pattern (Figure 1, bottom panel), but without major differences between the two generations tested (F0_EC10 = 58 and F1_EC10 = 57 mg Ag/kg).

In terms of reproduction, there was a clear dose-dependent inhibition with an increase from the first to the second generation (F1_EC50 = 988 and F2_EC50 = 234 mg Ag/kg soil, Table 1). This is clearly visible in the number of organisms over time at the concentrations above 100 mg Ag/kg (Figure 1C).

## 4. Discussion

In the present study, within the tested concentrations, survival was not affected, and reproduction was the most sensitive endpoint. This is in line with the effect of several chemicals/materials, including different NMs [7,9,46,47,48,49]. The toxicity increased ca. 4 times from the first to the second generation. Regarding size, the adult (although not juvenile) was affected in both generations. The population biomass, an approximation based on the size of the organisms times the number of organisms, was in fact the most sensitive endpoint, displaying a concentration-response pattern overall similar to that of the number of juveniles.

In terms of the standard OECD reproduction test [43], which corresponds to the results of the first-generation exposure, the reproduction EC50 (988 mg Ag/kg soil) was below the literature-reported values for other Ag NPs/NMs (see Table 2 for a relevant literature summary).

Considering the literature data, among the most tested silver materials, Ag NM300K was approximately 2 times more toxic to *F. candida* (reproduction EC50 = 540 mg Ag/kg) [7]. Such higher toxicity of Ag NM300K was also reported for other soil invertebrate species, for instance, for *E. crypticus* (reproduction EC50 = 161 mg Ag/kg soil for Ag NM300K [17] and 446 mg Ag/kg soil for Ag-Sigma [18], the same as tested here). Hence, comparing the relative toxicity between Ag NM300K and Ag-Sigma for the two species shows an approximately two-fold lower toxicity for Ag-Sigma for both species (see further below).

Other smaller (synthesized at 2.7 nm and 6.5 nm) Ag NPs showed higher toxicity to *F. candida* (exposed in artificial soil) than the Ag-Sigma, with reproduction EC50s of 159 and 206 mg Ag/kg for the 2.7 nm and 6.5 nm Ag NPs, respectively [9]. Despite differences in the tested soils, this could indicate a size-specific toxicity, i.e., higher toxicity for smaller Ag NPs. However, a study performed with 3–8 nm Ag NPs showed no reproductive toxicity to *F. candida* up to 673 mg Ag/kg soil (the highest tested concentration) [50], so size may not be the only determining factor, but toxicity may also depend on coating factors and more (the latter study [50] used paraffin-coated particles). Moreover, the toxicity of Ag-Sigma is lower than the reported for Ag ions, as in the literature data, with AgNO_3_ reproduction EC50 of 152 mg Ag/kg [7], or 159 mg Ag/kg soil [9], being lethal to *F. candida* (LC50 = 152 mg Ag/kg in LUFA 2.2 soil [7], and at LC50 = 98 mg Ag/kg in artificial soil [9]. Similarly to our results, there were no effects on collembolans’ survival [7,9,50].

Comparing the two soil model invertebrates, *F. candida* and *E. crypticus* (EC50 = 988 mg Ag/kg soil (current results) and EC50 = 446 mg Ag/kg soil [18], exposed to the same Ag-Sigma, the lower toxicity to the collembolans might be related to the different uptake routes for the groups: collembolans versus enchytraeids. Considering the ingestion route, the first consideration is that perhaps the mouth size could be a limiting factor. However, *F. candida* has a reported mouth opening of approximately 200–300 µm [51] and *E. crypticus* a mouth opening of approximately 100 µm [52]; hence, for neither species, this would not be a limiting factor. Second step, perhaps the dermal/cuticular (the outer and gut dermis) absorption route is the major differentiating factor. Although not studied in enchytraeids, the oral/gut uptake (i.e., gut dermis) of Ag NPs (50 nm, uncoated, powder) was quantified in the earthworm *Lumbricus rubellus* (also an Oligochaeta-like enchytraeids) to be 40 to 75% of the total Ag uptake [53], while the remaining occurs through outer dermis absorption. For collembolan species, there is no quantification of outer dermal versus oral uptake. However, the cuticle of collembolans and enchytraeids is fundamentally different in terms of structure and permeability (thicker, coated with chitin and lipids for rigidity and waterproofing in collembolans [54], and thin, flexible, and moist covered with mucus in enchytraeids [55]). Hence, the outer dermal uptake for enchytraeids is likely higher than for collembolans and hence contributes to the differences in toxicity.

Prolonged exposure of *F. candida* to Ag-Sigma for 56 days revealed an increase in toxicity in the second generation. Interestingly, for *E. crypticus*, the toxicity of Ag-Sigma did not increase with prolonged exposure (28d_EC50 = 446 and 56d_EC50 = 500 mg Ag/kg soil [18]). For *F. candida*, the increase in toxicity was ca. 4 times from the first to the second generation exposure (28d_EC50 = 988 and 56d_EC50 = 234 mg Ag/kg soil). Further, while for *E. crypticus*, Ag-Sigma had no effects on adults’ size (measured at day 28) [18], for *F. candida*, the size of the adults was significantly reduced following Ag-Sigma exposure for both 28 and 56 days. One hypothesis is that Ag-Sigma affects the growth of the collembolans, with possible effects on moulting. It has been shown that Ag NPs exposure caused developmental delays in the fruit fly *Drosophila melanogaster*, i.e., an increase in time required for the developing flies to reach a particular developmental stage, such as third instar larva, pupa, and adult [56,57], with interference on larval moulting [57]. The development and weight of *Podisus maculiventris* (a beneficial predatory insect in gardens and crop fields) were also inhibited by Ag NPs exposure [58]. Other NPs, such as zinc oxide (ZnO), have been shown to reduce moulting frequency in water crustacean *Daphnia pulex*, coupled with the down-regulation of the moulting-related gene *eip* (ecdysone-induced protein) [59]. *F. candida* grows by moulting continuously throughout its entire life cycle; hence, if Ag-Sigma interferes with the moulting process, it would affect not only growth (as observed in adults) but also cause a global developmental delay with further effects on reproductive output over time. In that case, the current one-generation standard tests are underestimating the potential effects of Ag NMs.

**Table 2:** Summary of relevant literature data on the ecotoxicity of silver (Ag) materials (AgNO_3_, AgNM300K, Ag-Sigma, Ag NPs 2.7 nm, Ag NPs 6.5 nm, Ag NPs 3–8 nm) on two model invertebrate species (*Folsomia candida*, *Enchytraeus crypticus*). Effect concentrations (LC50 (survival) and EC50 (reproduction)) represent the estimate in mg/kg soil DW. F1 and F2 are not available in all studies and represent the standard (28 days) versus longer exposure (56 days). n.e.: no effect.

Species	Ag Material	Soil	LC50	EC50 (F1/F2)	Reference
*F. candida*	Ag-Sigma	LUFA 2.2	n.e.	988/234	Current study
*F. candida*	AgNM300K	LUFA 2.2	n.e.	540	[7]
*E. crypticus*	Ag-Sigma	LUFA 2.2	1276	446/500	[19]
*E. crypticus*	AgNM300K	LUFA 2.2	675	161	[17]
*F. candida*	AgNO_3_	LUFA 2.2	179	152	[7]
*F. candida*	AgNO_3_	LUFA 2.2	284	100	[57]
*F. candida*	AgNO_3_	artificial OECD	97.97	126	[9]
*F. candida*	Ag NPs-2.7 nm	artificial OECD	n.e.	159	[9]
*F. candida*	Ag NPs-6.5 nm	artificial OECD	n.e.	206	[9]
*F. candida*	Ag NPs-3–8 nm	LUFA 2.2	n.e.	n.e. (>673)	[57]
*E. crypticus*	AgNO_3_	LUFA 2.2	75	62	[17]

Overall, current results showed an increase in toxicity in the second generation, which could not be predicted based on the standard test results alone. Moreover, that increase surpasses the toxicity of the reference Ag NM300K (28d_EC50 = 540 mg Ag/kg soil [7]).

## 5. Conclusions

The toxicity of the commercially available Ag NPs (Ag-Sigma, NPs < 150 nm, nanopowder) to *F. candida* was approximately half of that reported for the reference Ag material Ag NM300K, but increased (ca. 4 times) during a second-generation exposure. Effects were also observed on the adults’ size, suggesting that Ag-Sigma might interfere with the moulting of the animals. It was shown that the toxicity observed for the second-generation exposure was not predicted based on the standard test results. Hence, prolonged exposures are highly recommended to improve the risk assessment of Ag NMs/NPs and (likely) other NMs to the soil compartment. Further studies should focus on the coverage and understanding of the long-term impacts (behaviour and transformation) of Ag NPs in the soil compartment for the improved understanding of their risks.

## Figures and Tables

**Table 1:** Summary of the effect concentrations (ECx with 95% confidence intervals—CI), expressed as mg Ag per kg soil (dry weight), *Folsomia candida* exposed to Ag-Sigma, in LUFA 2.2 soil. The models used are Logistic 2 parameters (Log2P) or threshold sigmoid 2 parameters (Thres2P). S: slope; Y0: top point; n.e.: no effect.

Endpoint	Generation/ Time (Days)	EC10 (95% CI)	EC50 (95% CI)	EC90 (95% CI)	Model and Parameters
Survival	F0/28	n.e.	n.e.	n.e.	-
Reprod.	F1/28	33 (9–115)	988 (567–1721)	8098 (1970–33,286)	Thres2P (S: 0.37; Y0: 860.8; r^2^: 0.7)
Size (adults)	F0/28	58 (10–346)	>>3200	>>3200	Thres2P (S: 0.15; Y0: 1.4; r^2^: 0.5)
Size (juveniles)	F1/28	n.e.	n.e.	n.e.	-
Biomass (total population)	F0 + F1/28	4 (1–26)	622 (340–1138)	>>3200	Log2P (S: 0.25; Y0: 92; r^2^: 0.8)
Survival	F1/56	n.e.	n.e.	n.e.	-
Reprod.	F2/56	34 (15–80)	234 (161–340)	1591 (691–3664)	Log2P (S: 0.66; Y0: 817.3; r^2^: 0.8)
Size (adults)	F1/56	57 (12–259)	>>3200	>>3200	Thres2P (S: 0.27; Y0: 1.4; r^2^: 0.4)
Size (juveniles)	F2/56	n.e.	n.e.	n.e.	-
Biomass (total population)	F1 + F2/56	6 (2–22)	165 (107–256)	4318 (1427–13,063)	Log2P (S: 0.39; Y0: 101; r^2^: 0.9)

## Data Availability

The original contributions presented in this study are included in the article. Further inquiries can be directed to the corresponding author.
